# A scoring system basing pathological parameters to predict regional lymph node metastasis after preoperative chemoradiotherapy for locally advanced rectal cancer: implication for local excision

**DOI:** 10.18632/oncotarget.10965

**Published:** 2016-07-30

**Authors:** Xiao-Jie Wang, Pan Chi, Hui-Ming Lin, Xing-Rong Lu, Ying Huang, Zong-Bin Xu, Sheng-Hui Huang, Yan-Wu Sun, Dao-Xiong Ye, Qian Yu

**Affiliations:** ^1^ Department of Colorectal Surgery, Union Hospital, Fujian Medical University, Fuzhou, Fujian 350001, People's Republic of China; ^2^ Department of Pathology, Union Hospital, Fujian Medical University, Fuzhou, Fujian 350001, People's Republic of China

**Keywords:** rectal cancer, preoperative chemoradiotherapy, regional nodal metastasis

## Abstract

Local excision is an alternative to radical surgery that is indicated in patients with locally advanced rectal cancer (LARC) who have a good response to chemoradiotherapy (CRT). Regional lymph node status is a major uncertainty during local excision of LARC following CRT. We retrospectively reviewed clinicopathologic variables for 244 patients with LARC who were treated at our institute between December 2000 and December 2013 in order to identify independent predictors of regional lymph node metastasis. Multivariate analysis of the training sample demonstrated that histopathologic type, tumor size, and the presence of lymphovascular invasion were significant predictors of regional nodal metastasis. These variables were then incorporated into a scoring system in which the total scores were calculated based on the points assigned for each parameter. The area under the curve in the receiver operating characteristic analysis was 0.750, and the cutoff value for the total score to predict regional nodal metastasis was 7.5. The sensitivity of our system was 73.2% and the specificity was 69.4%. The sensitivity was 77.8% and the specificity was 51.2% when the scoring system was applied to the testing sample. Using this system, we could accurately predict regional nodal metastases in LARC patients following CRT, which may be useful for stratifying patients in clinical trials and selecting potential candidates for organ-sparing surgery following CRT for LARC

## INTRODUCTION

Local excision (LE) is an acceptable treatment for early-stage rectal cancer (T1) [1]. This organ-sparing surgery avoids a permanent stoma in patients who would otherwise require abdominoperineal resection (APR). LE is advantageous in that it better preserves anorectal, sexual, and urinary functions compared to anterior resection (AR) [2].

The metastatic status of mesorectal lymph nodes may be an important determinant of local and distant recurrent risk after LE [2]. The reported rate of lymph node metastasis in early-stage rectal cancer (T1) ranges from 6 to 11% [3]. However, less is known about the incidence of metastatic lymph node involvement in locally advanced rectal cancer (LARC, cT3-4 Nx or cTx N+) following a good response of the primary tumor to neoadjuvant treatment. Park et al. [4] reported that the risk of residual mesorectal lymph node metastasis was high despite a good response to neoadjuvant chemoradiotherapy (CRT) within the bowel wall: 20.8% among ypT2, 17.1% among ypT1, and 9.1% among ypT0 patients. Thus, the use of ypT staging to stratify patients for LE may not be appropriate [4, 5]. Importantly, no studies have demonstrated a relationship between the histopathologic features of the primary residual tumor and the metastatic status of regional lymph nodes.

Whether LE is indicated for LARC patients who have a good response to CRT is controversial. Several small studies of patients with LARC who underwent CRT followed by full-thickness LE have demonstrated long-term oncological results similar to those achieved by CRT followed by APR or AR. However, the indications for LE varied in different studies [2, 6–8]. Therefore, additional clinical studies with standardized inclusion criteria are required.

The first aim of this study was to investigate whether the histopathologic features of the primary tumor following CRT correlated with the metastatic status of regional lymph nodes. The second aim was to identify preoperative predictive factors of regional nodal metastasis based on pathologic findings, and to develop a scoring system for the prediction of regional nodal status that could aid decision-making if LE is indicated. The third aim was to investigate the prognostic impact of positive regional nodes in LARC patients following CRT and curative resection.

## RESULTS

### Patient clinicopathological characteristics

The clinicopathological characteristics for all 244 patients are shown in Table 1. Gender, age, tumor distance from the anal verge, ypTNM stage, preoperative serum CEA level, and preoperative serum CA199 level were comparable between the training and testing cohorts (*P* > 0.05). Of the 126 patients in the training sample, 81 (64.3%) were men and 45 (35.7%) were women. The mean age at diagnosis was 53.7 years (standard deviation [SD]: 14.3 years). There were 41 patients (32.5%) with regional lymph node metastasis (ypN+) and 85 patients (67.5%) with no regional nodal involvement (ypN-). We identified 21 patients (16.7%) in the database who had a complete pathologic response (ypCR) following CRT and surgery. Of these patients, 1 (4.8%) had confirmed lymph node involvement. The incidence of lymph node involvement was 38.1% (40/105) among patients with residual disease. The patient clinicopathological data were incorporated into a univariate analysis (Table 2). The clinical characteristics of the patients, which included gender, age, tumor distance from the anal verge, preoperative serum CEA levels, preoperative serum CA199 levels, pre-CRT clinical T stage, post-CRT clinical T stage (ycT stage), and chemotherapy regimens, were similar between the ypN+ and ypN- patients (*P* > 0.05).

**Table 1:** The clinicopathological characteristics of patients with LARC following CRT

	Training sample *(n* = 126)	Testing sample (*n* = 118)	*P* value
Gender			0.984
Male	81 (64.3)	76 (64.4)	
Female	45 (35.7)	42 (35.6)	
Age (years)	53.7 ± 14.3	55.3 ± 10.8	0.349
Tumor distance to anal verge (cm)	5.8 ± 2.5	5.4 ± 1.7	0.194
Pathologic T stage (ypT)			0.083
T0–T2	48 (38.1)	57 (49.1)	
T3–T4	78 (61.9)	59 (50.9)	
ypTNM stage			0.185
I	40 (31.7)	50 (42.4)	
II	45 (35.7)	32 (27.1)	
III	41 (32.5)	36 (30.5)	
Preoperative serum CEA levels (ng/ml)	3.8 ± 8.5	4.6 ± 8.6	0.452
Preoperative serum CA199 levels (U/ml)	18.4 ± 28.4	18.0 ± 28.2	0.911

**Table 2:** The univariate analysis of predictors for metastatic status of regional nodes

	ypN- (*n*= 85)	ypN+ (*n* = 41)	*P* value
Gender			0.514
Male	53 (65.4)	28 (34.6)	
Female	32 (71.1)	13 (28.9)	
Age (years)	54.0 ± 17.0	53.6 ± 13.0	0.889
Tumor size (cm)	2.8 ± 1.4	3.8 ± 2.0	0.001
Tumor distance to anal verge (cm)	5.9 ± 2.8	5.8 ± 1.8	0.765
Preoperative serum CEA levels (ng/ml)	3.6 ± 9.3	4.1 ± 6.6	0.732
Preoperative serum CA199 levels (U/ml)	18.8 ± 32.1	18.2 ± 26.6	0.912
Gross type^a^			0.068
Ulcerative	69 (70.4)	29 (29.6)	
Infiltrative	8 (72.7)	3 (27.3)	
Expanding	2 (28.6)	5 (71.4)	
Tumor differentiation^b^			0.001
Well-moderately differentiated	66 (77.6)	19 (22.4)	
Poorly differentiated, others^c^	19 (48.7)	20 (51.3)	
Histopathologic type			<0.001
Adenocarcinoma	72 (75.8)	23 (24.2)	
Mucinous or signet ring cell adenocarcinoma	13 (41.9)	18 (58.1)	
Tumor regression grade			0.555
I	60 (65.9)	31 (34.1)	
II-III	25 (71.4)	10 (28.6)	
Pre-CRT clinical T stage			0.501
T3	30 (71.4)	12 (28.6)	
T4	55 (65.5)	29 (34.5)	
Post-CRT clinical T stage (ycT)			0.194
T1	3 (100)	0 (0)	
T2	6 (100)	0 (0)	
T3	21 (63.6)	12 (36.4)	
T4	55 (65.5)	29 (34.5)	
Pathologic T stage (ypT)			0.013*
T0	20 (95.2)	1 (4.8)	
T1	4 (66.7)	2 (33.3)	
T2	16 (76.2)	5 (23.8)	
T3	25 (53.2)	22 (46.8)	
T4	20 (64.5)	11 (35.5)	
T downstaging			0.422
Yes	52 (61.2)	22 (53.7)	
No	33 (38.8)	19 (46.3)	
Neural invasion	1 (1.2)	0 (0)	1.000*
Mesenteric tumor nodules	1 (1.2)	0 (0)	1.000*
Lymphovascular invasion	1 (1.2)	4 (9.8)	0.038*
Chemotherapy regimen			0.460
Capox	69 (69.7)	30 (30.3)	
Folfox	5 (50.0)	5 (50.0)	
Capecitabine	8 (72.7)	3 (27.3)	
Others	3 (50.0)	3 (50.0)	

a With data missing of 10 cases (7.9%)

b With data missing of 2 cases (1.6%)

c Included mucinous and signet ring cell carcinoma

* Fisher's exact test

### Gross pathologic assessment of the primary tumor

**Figure 1:**
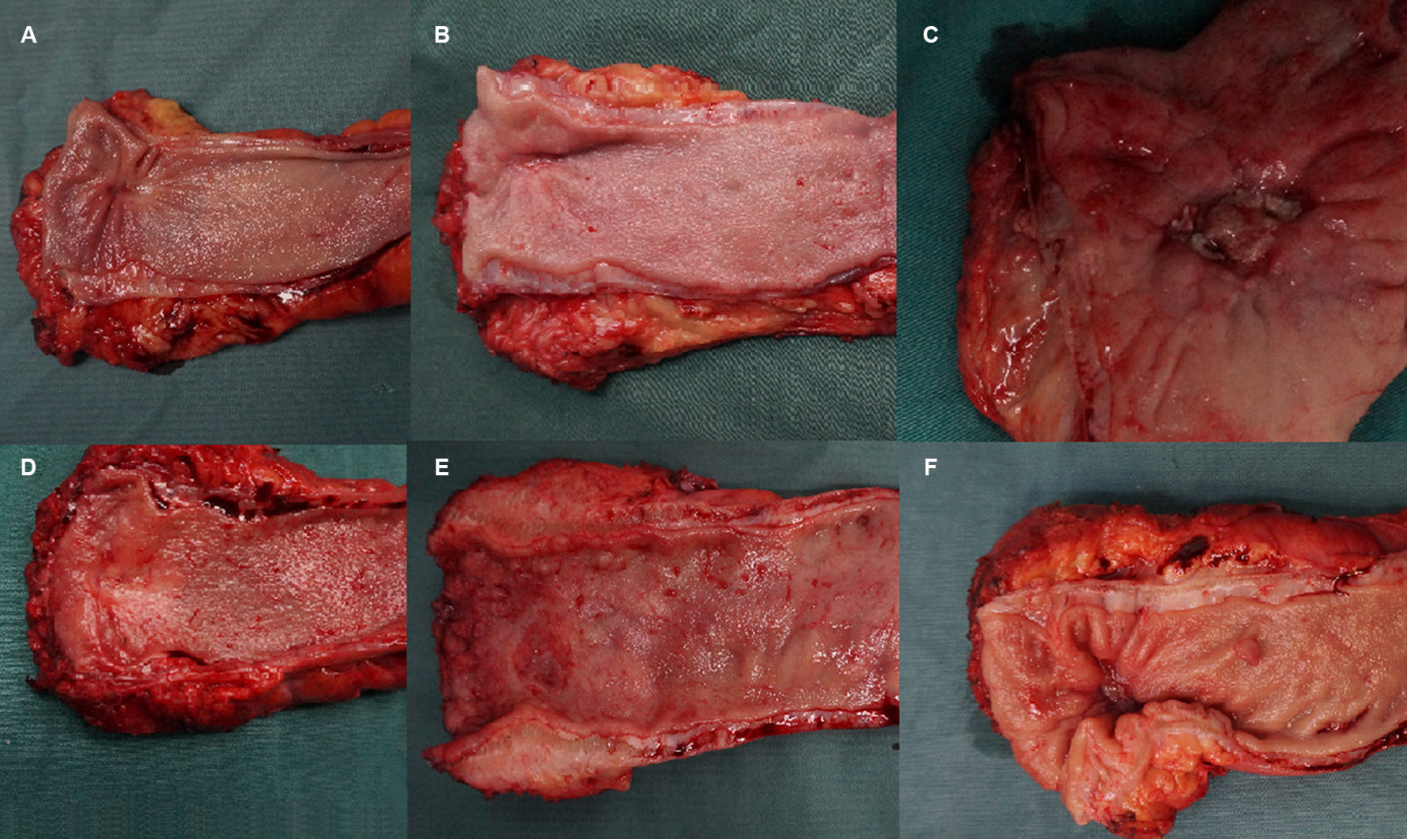
Digital photographs demonstrating the similarities between resected primary tumor specimens after CRT with or without regional nodal metastasis (**A**) ypT2N0; (**B**) ypT0N0; (**C**) ypT3N0; (**D**) ypT2N1; (**E**) ypT3N1; (**F**) ypT2N1.

Univariate analysis demonstrated that the gross appearance of the primary tumor that remained after CRT was similar between ypN- and ypN+ patients (*P* = 0.068). We analyzed photographs of resected primary tumor specimens with or without regional nodal metastasis. In some cases, gross assessment of the primary tumors with regional nodal metastasis revealed either flat fibrotic scars or deep ulcerations with central necrotic regions. It was frequently impossible to distinguish between samples with and without regional nodal metastasis based on the gross appearance alone (Figure 1). However, ypN+ patients generally had much larger tumors than ypN- patients (3.8 cm vs. 2.8 cm, respectively, *P* = 0.001) (Table 2). A significant association between ypN+ status and larger primary tumor size was also observed (*P* = 0.017).

### Histopathologic assessment of the primary tumor

**Figure 2:**
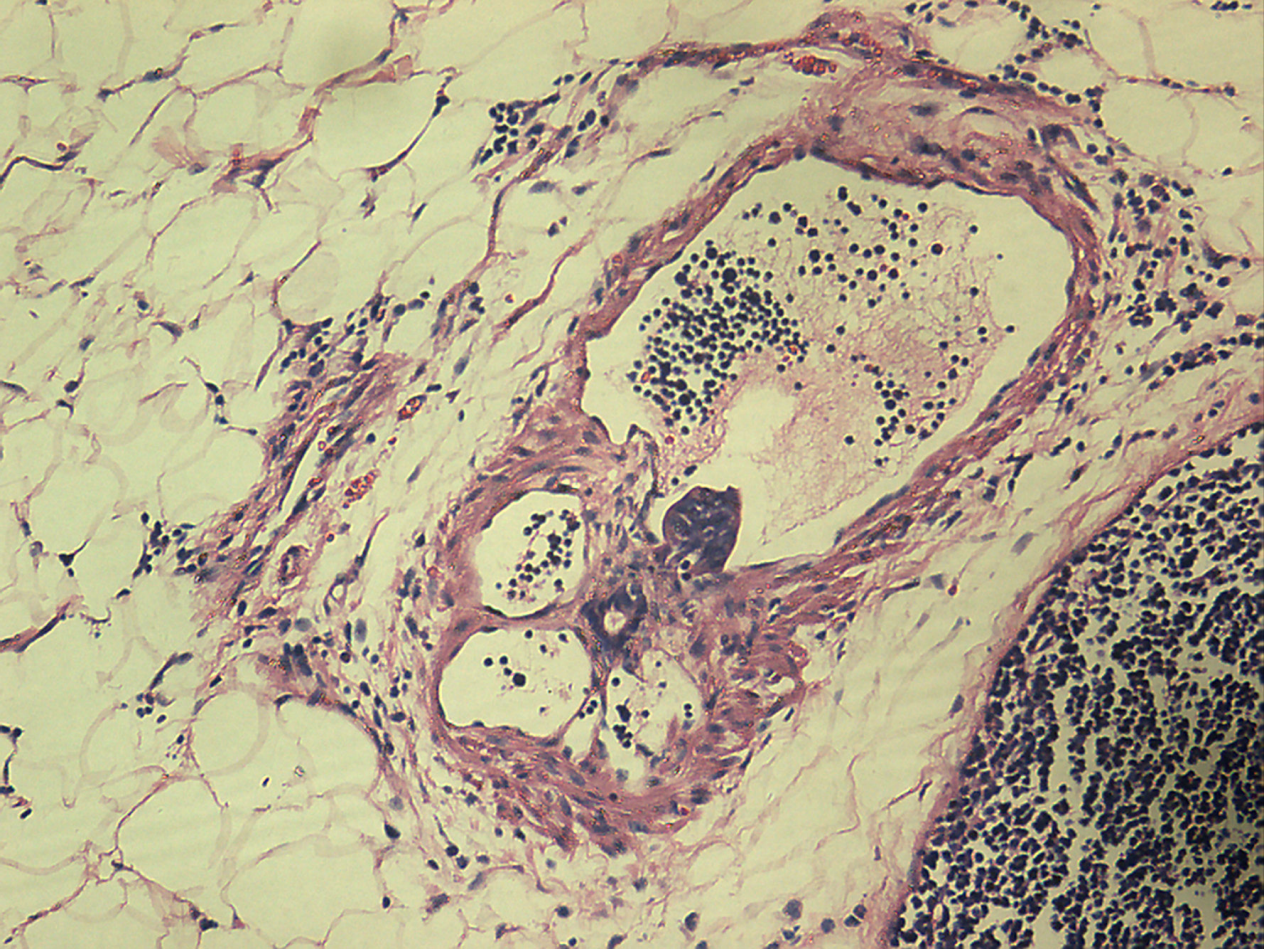
Characteristic histological features of vascular invasion (Hematoxylin and eosin (H.E.) staining, 200× magnification) Tumor emboli were observed in vascular spaces.

**Figure 3:**
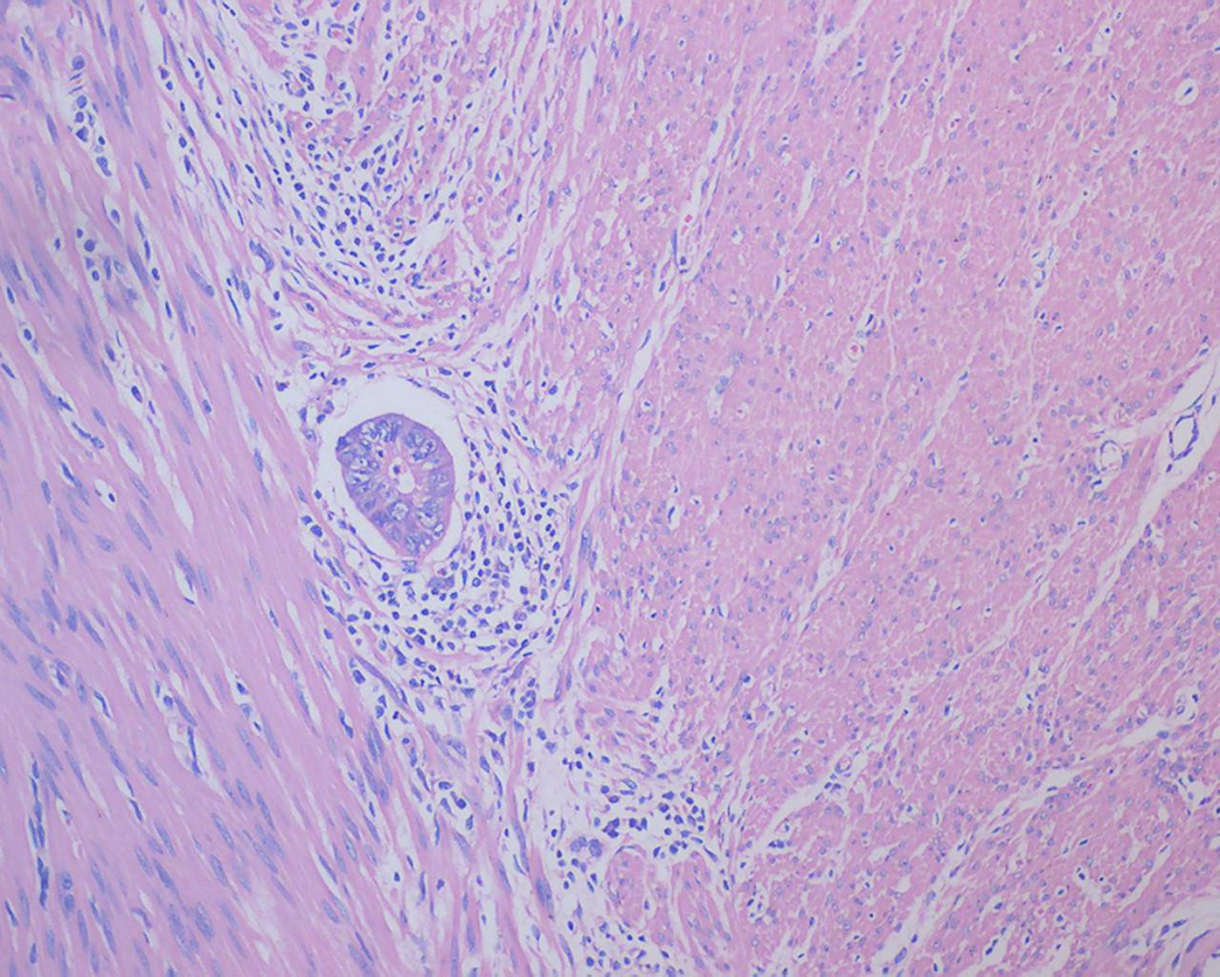
Characteristic histological features of lymphatic invasion (H.E. staining, 200× magnification) Tumor emboli were observed in lymphatic vessels.

**Figure 4:**
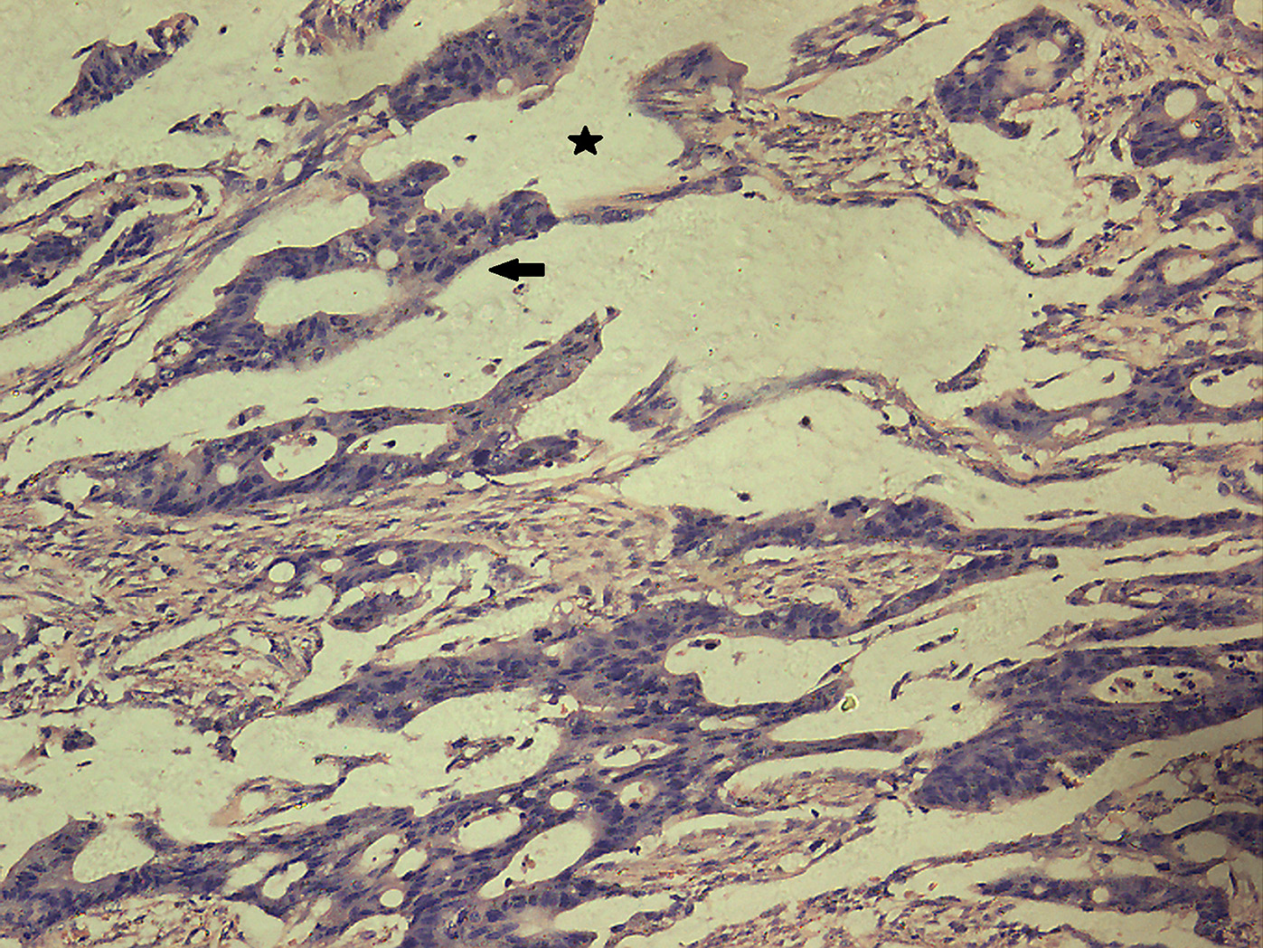
Characteristic histological features of mucinous adenocarcinoma (H.E. staining, 200× magnification) Extracellular mucinous lakes (star) and clusters of mucinous cancer cells (arrow).

**Figure 5:**
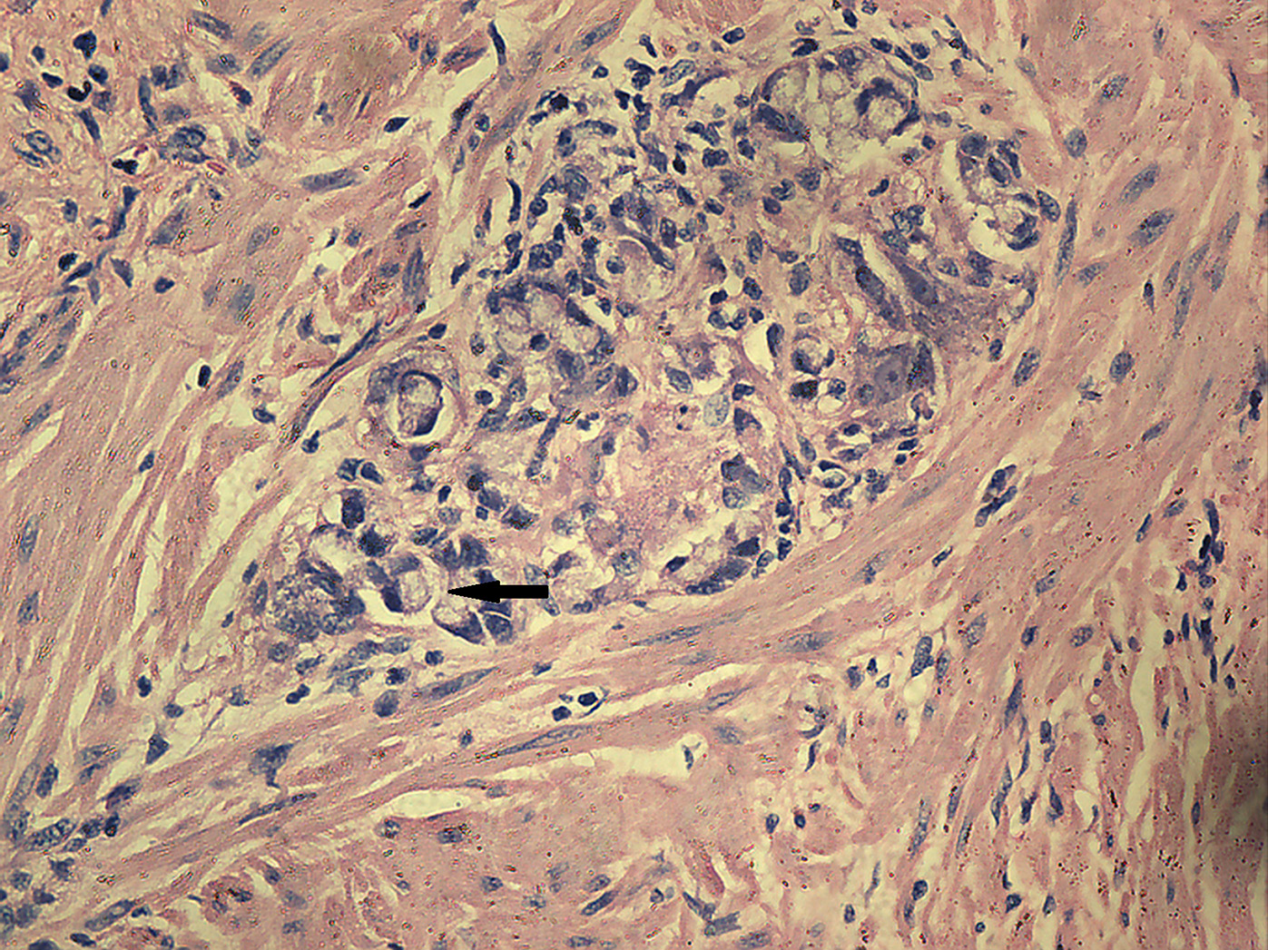
Characteristic histological features of signet ring cell adenocarcinoma (H.E. staining, 200× magnification) The arrow indicates a signet ring cell adenocarcinoma.

Univariate analysis showed no differences in the tumor regression grade, incidence of T downstaging, neural invasion, or mesenteric tumor nodules (*P >* 0.05) between the ypN+ and ypN− groups. The ypN+ patients had significantly less differentiated tumors (*P* = 0.001), higher ypT stage (*P* = 0.013), and a higher incidence of lymphovascular invasion (9.8% vs. 1.2%, *P* = 0.038) than ypN− patients (Figures 2–3). In addition, mucinous or signet ring cell adenocarcinomas (poorly differentiated histological subtypes) were more commonly observed in ypN+ patients than in ypN- patients (*P <* 0.001) (Table 2, Figures 4–5).

### Predictive scoring system for regional nodal involvement

**Figure 6:**
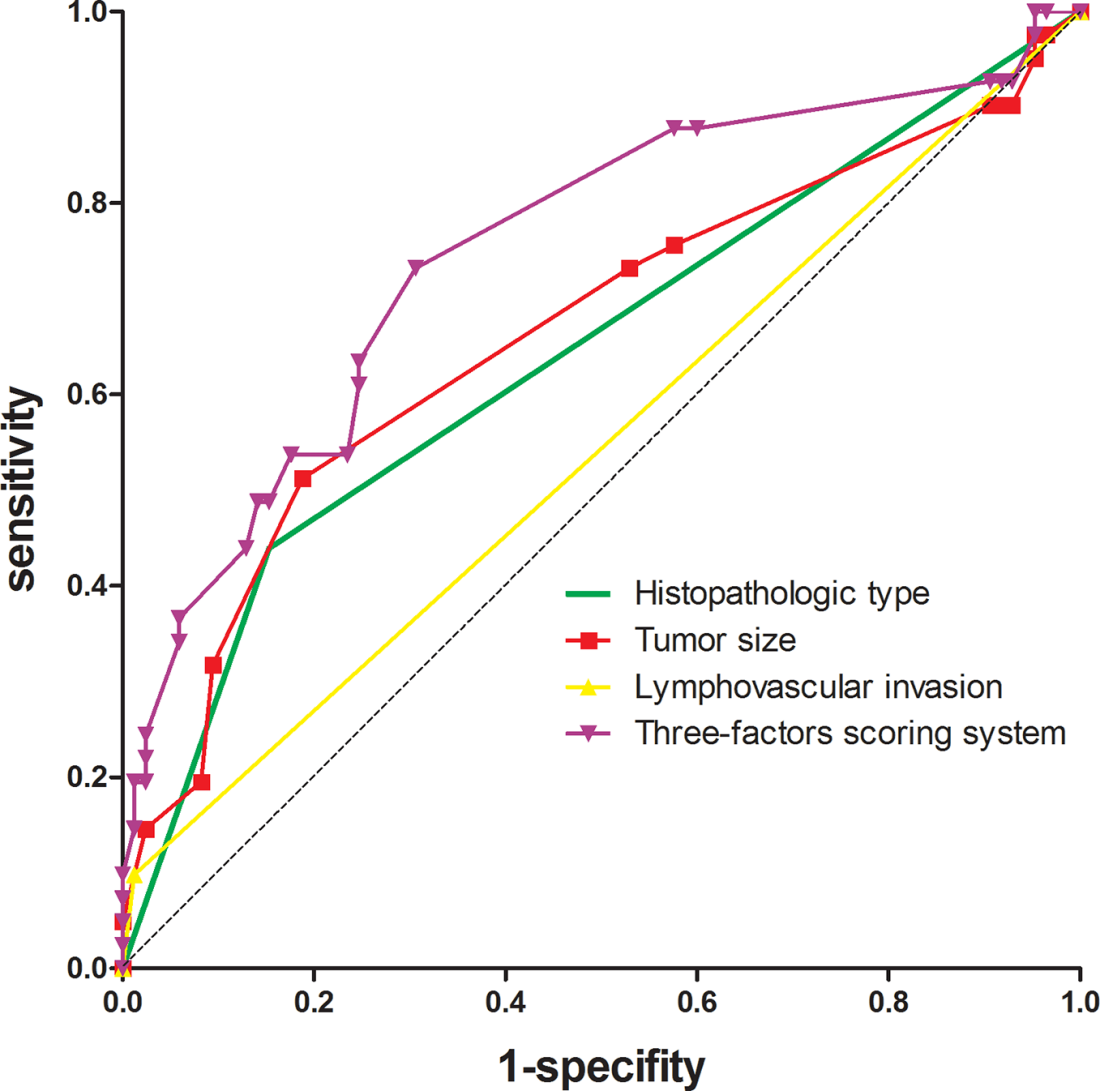
ROC curves of the three predictors and the scoring system that was developed to predict regional lymph node involvement

**Figure 7:**
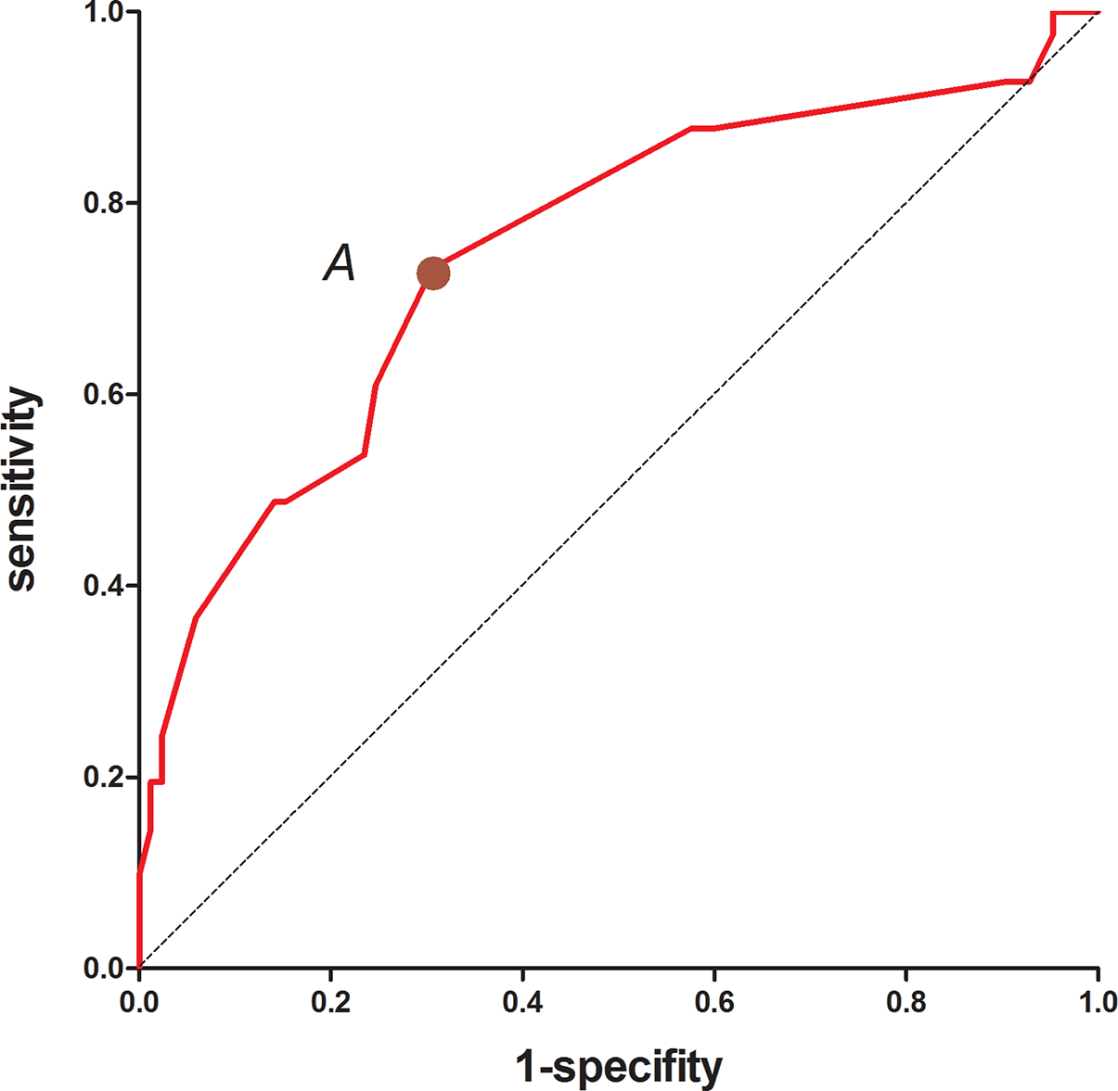
ROC curve of the total scores The optimal cutoff point A (7.5) on the ROC curve provided the best predictive ability.

To identify predictors of the metastatic status of regional nodes, multivariate analysis was performed using variables that were found to be significant in univariate analysis (e.g. tumor size, differentiation, lymphovascular invasion, ypT stage, and histopathologic type). Among the various factors, the histopathologic type (odds ratio [OR], 3.923; 95% confidence interval [CI], 1.577–9.760, *P* = 0.003), tumor size (OR, 1.381; 95% CI, 1.071–1.781, *P* = 0.013), and lymphovascular invasion (OR, 10.964; 95% CI, 1.092–110.083, *P* = 0.042) were independently correlated with the metastatic status of regional lymph nodes (Table 3). To evaluate the predictive power of the various factors, we performed receiver operating characteristic (ROC) curve analyses and calculated Youden's index. We found that tumor size was the strongest predictor of regional nodal metastasis, which had a sensitivity, specificity, positive predictive value (PPV), and negative predictive value (NPV) of 51.2%, 81.2%, 74.2%, and 60.2%, respectively (Table 4). The area under the ROC curve was 0.664. The highest predictability was achieved using a combination of these three predictors (sensitivity of 73.2%, specificity of 69.4%, PPV of 70.5%, and NPV of 72.1%). The area under the ROC curve was 0.750 (Table 4, Figure 6). These three predictors were therefore incorporated into a scoring system by assigning points to the various variables based on their coefficients in the logistic analysis. One point was added for tumor size per centimeter, four points were added for adenocarcinoma, eight for mucinous or signet ring cell adenocarcinoma, and seven for lymphovascular invasion (Table 5). The scores were calculated by taking the sum of the points from all predictors. We performed ROC curve analyses on the scores and determined that a cutoff value of 7.5 points had the best predictability (Figure 7). We next applied the scoring system developed in the training sample to an independent testing cohort consisting of 118 patients in order to evaluate the predictive power of the system. Using this approach, we achieved a sensitivity of 77.8% and specificity of 51.2%.

**Table 3:** Logistic regression analysis of predictors for metastatic status of regional nodes

Factors	regression coefficient	SE	Wald	*P* value	odd ratio	95%CI
Histopathologic type						
Adenocarcinoma / mucinous or signet ring cell adenocarcinoma	1.367	0.465	8.642	0.003	3.923	1.577–9.760
Tumor size (cm)	0.323	0.130	6.201	0.013	1.381	1.071–1.781
Lymphovascular invasion	2.395	1.177	4.140	0.042	10.964	1.092–110.083
Constant	−3.712	0.759	23.902	0.000	0.024	

**Table 4:** ROC curve analyses of predictors of regional nodal metastasis

	Sensitivity	Specifity	PPV	NPV	AUC	*P* value
Histopathologic type	43.9%	84.7%	74.2%	60.2%	0.643	0.009
Tumor size	51.2%	81.2%	73.1%	62.5%	0.664	0.003
Lymphovascular invasion	9.8%	98.8%	52.3%	89.1%	0.543	0.436
Combined predictors	73.2%	69.4%	70.5%	72.1%	0.750	<0.001

PPV, positive predictive value

NPV, negative predictive value

AUC, area under the curve

**Table 5:** Scoring system to predict regional lymph nodes metastasis

Predictors	Score
Histopathologic type	
Adenocarcinoma	4
Mucinous or signet ring cell adenocarcinoma	8
Tumor size	Long axis diameter of tumor in cm×1
Lymphovascular invasion	7

### Long-term outcomes

**Figure 8:**
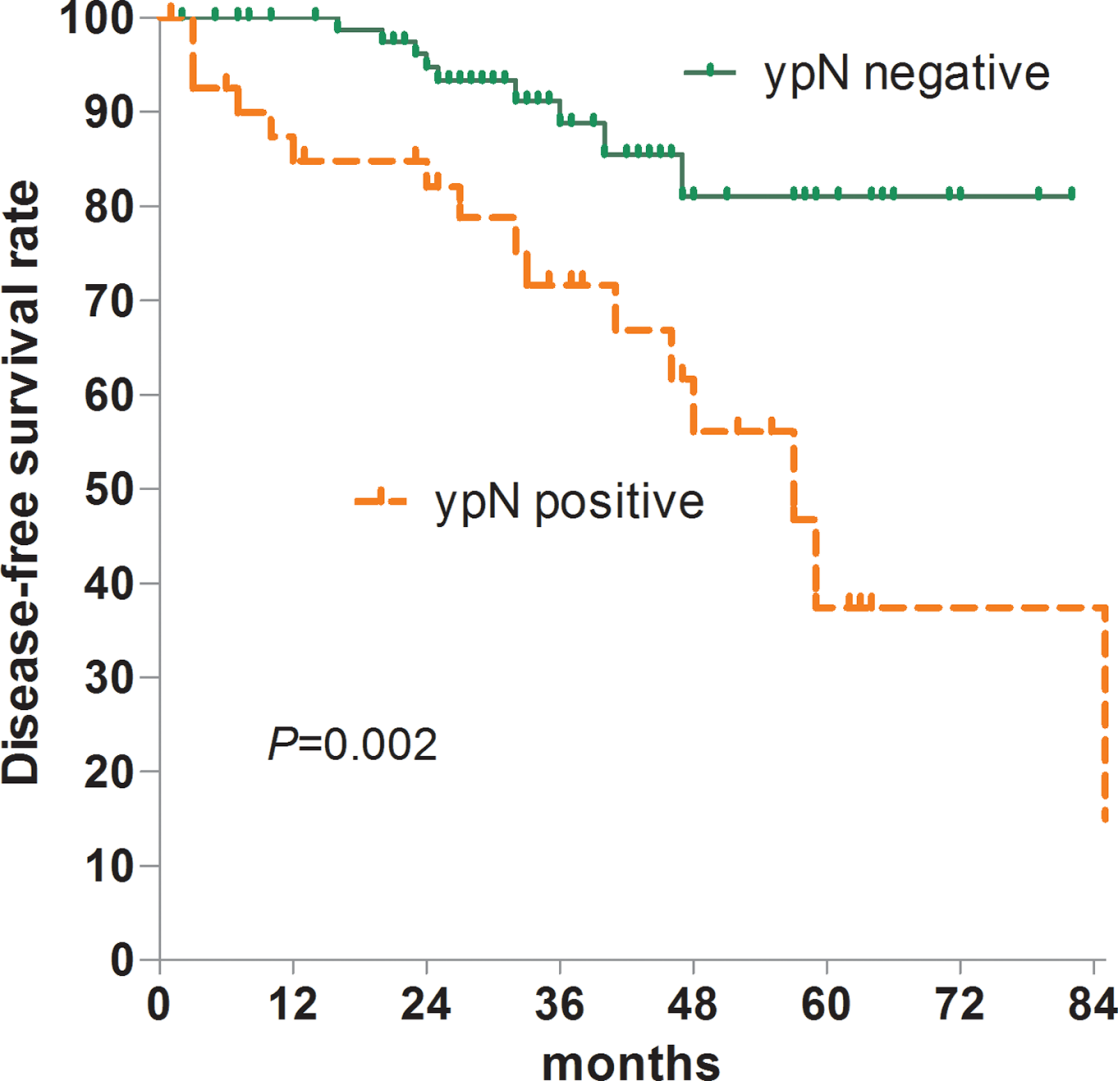
DFS according to regional lymph nodal status

The median follow-up for was 33 months (range, 1–99 months), and the 5-year disease-free survival (DFS) rate was 64.1%. Recurrence was observed in 16 patients. One patient had local recurrence, 14 had systemic, and one had both local and systemic. Overall, the 5-year DFS was 81.0% among ypN− patients and 37.4% among ypN+ patients (*P* = 0.002) (Figure 8). Multivariate Cox regression analysis demonstrated that regional lymph node metastasis was the only independent factor associated with an unfavorable 5-year DFS rate (OR, 3.504; 95% CI, 1.530–8.024, *P* = 0.003).

## DISCUSSION

One of the major uncertainties when performing LE for LARC following CRT is the status of regional lymph nodes. Reluctance to adopt LE for LARC following CRT is primarily due to concerns about leaving behind positive regional lymph nodes, especially in the light of data indicating that positive regional lymph nodes that remain after CRT are the only independent poor prognostic factor for 5-year DFS [9]. Several studies have reported that recurrence following LE with neoadjuvant CRT was associated with lymph node metastasis [10]. In our study, a relatively high percentage of patients (32.5%) had regional nodal metastasis.

No imaging techniques can reliably predict the status of regional lymph nodes following CRT without definitive resection. According to a recent meta-analysis, there was no significant difference between the sensitivity estimates for magnetic resonance imaging (MRI) (61.8%) and endoluminal ultrasound (49.8%) for restaging of lymph node involvement in LARC patients following CRT [11]. To date, there is limited data on the relationship between the gross appearance/histology of tumors after CRT and regional lymph node status. A recently published study based on the Surveillance, Epidemiology, and End Results Program (SEER) database showed that ypT0–1 combined with pre-CRT MRI staging cN0 might be useful for selecting LARC patients who would be good candidates for LE after CRT. However, no practical criteria for the diagnosis of cN0 were established in the study. Moreover, several important histological characteristics such as tumor differentiation and lymphovascular invasion were not included in the analysis due to a lack of information, and multivariate analysis was not performed to adjust for confounding factors [12].

Here, we determined that histopathologic type, tumor size, and lymphovascular invasion were independent predictors of regional nodal metastasis in a multivariate analysis. Additionally, we developed a scoring system based on these three pathologic parameters in order to predict the metastatic status of regional lymph nodes in LARC patients after CRT. The scoring system had a sensitivity of 73.2%, specificity of 69.4%, PPV of 70.5%, and NPV of 72.1%. In the validation analysis, we achieved 77.8 % sensitivity and 51.2% specificity.

Our system has several potential applications. First, it may act as a tool to assist with treatment selection. Because radical surgery is associated with significant morbidity and mortality, patients who have prohibitive comorbidity with a score greater than 7.5 points may benefit from LE. In several small retrospective studies, patients with pretreatment T3 rectal cancer who underwent CRT followed by full-thickness LE had local recurrence rates that were similar to those achieved with CRT followed by total mesorectal excision (TME) [8, 13]. Since some pathologic parameters were not precisely available prior to surgery for biopsy specimens. This could have resulted in underestimation of the total score due to the superficial biopsy of the tumors. Therefore, if LE is performed on the primary tumor after CRT, based on predicted scores calculated from biopsy specimens, a detailed histological evaluation of the resected specimen should be performed. If lymph node involvement is predicted, further treatment with radical surgery or irradiation may be necessary. Second, this scoring system may be useful for designing clinical trials (particularly for patient stratification). The lack of standardization in patient selection for LE was a confounding factor in previous studies. The baselines of the LE and TME groups were generally not comparable. LE patients typically have higher baseline comorbidity and smaller tumors that are closer to the anal verge [8, 13]. Therefore, randomized clinical trials (RCTs) with standardized patient selection criteria are necessary.

We found that tumor size was the strongest predictor of regional nodal metastasis. Previous studies have suggested that primary tumor size is a predictor of tumor response to CRT [14]. Several studies, including two RCTs, reported that reduction of the tumor to 2–3 cm was usually required in order for patients to qualify for LE [2, 6, 7]. In addition, the diameters of residual mucosal abnormalities were strongly correlated with ypT stage. One study showed that 95.2% (40/42) of tumors downstaged to ypT0/1 had residual mucosal abnormalities with diameters of 3 cm or less after CRT [15]. To date, little is known about the association between primary tumor size after CRT and regional nodal disease in LARC patients. We demonstrated a significant relationship between ypN positivity category and primary tumor size in univariate and multivariate analyses. Tumor size is another factor that must be considered, as tumors must be relatively small in order for transanal resection to be performed. Tumor size is usually easy to estimate prior to surgery and might be an excellent predictor of regional nodal disease. Photographs of resected specimens of primary tumors with or without regional nodal metastasis were also analyzed in this study. In many cases, we were unable to distinguish tumors with regional nodal metastasis from those without based on gross appearance alone.

Histological analysis of residual primary tumors resulted in several findings that may be clinically significant. First, we demonstrated that mucinous and signet ring cell adenocarcinomas, two poorly differentiated histopathologic types, were useful predictors of regional nodal metastasis. Poor tumor differentiation was associated with nodal involvement in early rectal cancer, and tumor differentiation in LARC patients was proposed to be a biomarker that could be used to predict tumor response to CRT [16, 17]. Mucinous adenocarcinoma was shown to have a poor response to CRT, which manifested as larger residual tumors, a higher incidence of margin positivity, and a high rate of residual nodal metastasis [18]. Qiu et al. [19] reported that poor differentiation and T4 stage resulted in a high incidence of CRT resistance. However, patients with signet ring cell carcinoma exhibited either a complete histological response or no response [20]. Thus, additional studies are required to assess the role of histopathologic type in predicting nodal involvement. Lymphovascular invasion was also associated with mesorectal nodal metastases after CRT, which was consistent with data from previous studies [15]. In our study, lymphovascular invasion had a specificity of 98.8% and may therefore be beneficial for selecting patients who are unsuitable for LE. Lymphovascular invasion was also associated with poor survival after CRT [21].

Our study had several limitations. First, it was a retrospective study and some missing data could not be reconciled. However, the data used were collected prospectively in a highly standardized manner, and the final data set was more than 90% complete. To further improve the accuracy, a prospective study with standardized pathologic diagnostic criteria is necessary. Second, some clinical information such as the MRI or transrectal ultrasonography findings [22, 23] was not available and therefore was not included in our analysis. These data will be included in future studies in order to determine whether they could improve the predictability of the scoring system.

In conclusion, our results confirm that LARC patients with regional nodal metastasis after CRT have a poor prognosis. Tumor size, histological type, and lymphovascular involvement were significant predictors of the metastatic status of regional lymph nodes. A scoring system that could predict regional nodal involvement based on these factors was therefore established. This system has relatively high sensitivity and specificity, and may assist clinicians with the selection of the optimal surgical strategy.

## MATERIALS AND METHODS

### Patients

We selected 1,049 consecutive patients from the colorectal cancer database at our institution who were diagnosed and treated for rectal cancer in the Department of Colorectal Surgery of Affiliated Union Hospital of Fujian Medical University between December 2000 and December 2013. The inclusion criteria for this study were as follows: histologically proven rectal adenocarcinoma, clinical locally advanced rectal cancer (cT3-4 Nx or cTx N+), received neoadjuvant treatment.

Of these patients, 244 fulfilled the inclusion criteria for the study. Patients who had stage I (*n* = 143) or stage IV (*n* = 117) disease, who did not receive neoadjuvant treatment (*n* = 449), who only received short-course radiotherapy or preoperative chemotherapy (*n* = 12), or who underwent palliative resection or emergency surgery (*n* = 84) were excluded from the study.

Before CRT, all patients were evaluated by staging workups, which included a digital rectal examination, video colonoscopy, chest radiography, transrectal ultrasonography, abdominopelvic computed tomography (CT), and pelvic MRI. After CRT, the same staging workups were performed (within a week) before surgery to evaluate the response to CRT. All patients received 5-fluorouracil-based preoperative concurrent chemotherapy. Preoperative radiotherapy was delivered to the whole pelvis at a dose of 45 Gy in 25 fractions, followed by a 5.4 Gy boost in three fractions within 6 weeks. Surgery was recommended 6 to 8 weeks after the completion of preoperative radiotherapy. Standard surgical resection was performed for all patients. TME was performed for patients with middle and low rectal cancers, and partial mesorectal excision with a distal margin of at least 5 cm was performed for high rectal cancers. The clinical and pathologic stages were determined according to the 7th edition of the American Joint Committee on Cancer TNM staging system [24].

Gross pathologic assessment included tumor size (measured by the long axis diameter of the tumor in centimeters) and the gross type of primary tumor that remained after CRT. This process was aided by the inspection of digital photographs of the endoluminal aspects of the resected tumor specimens before formalin fixation (when available). Histopathological assessment included the histopathologic type, tumor differentiation, tumor regression grade, mesenteric tumor nodules, lymphovascular and neural invasion, T-downstaging, ypT stage, and ypN stage. The evaluation of T-downstaging was based on a comparison between previous clinical staging and the results of the pathologic evaluation. Patients received postoperative follow-up every 3 months for the first 2 years and annually thereafter. At each visit, imaging studies including chest radiography and abdominopelvic MRI were performed. Colonoscopy was performed 3 months to 1 year after the initial surgery and then every year thereafter.

### Statistical analysis

To develop and validate a predictive scoring system, we assigned 126 of the 244 patients who were treated between December 2000 and December 2011 to the training sample. An independent cohort of 118 patients who were treated between January 2012 and December 2013 were assigned to the testing sample. All statistical analyses were performed using the SPSS software (ver. 17 SPSS Inc., Chicago, IL, USA). Categorical variables were compared using chi-squared and Fisher's exact tests. Continuous variables were compared using Student's *t* tests. Logistic regression was performed on the training sample to identify pathologic predictors of regional nodal metastasis. Variables that were significantly correlated with regional nodal metastasis (*p* < 0.05) were entered into a logistic regression model using a forward selection method. These predictors were then incorporated into a scoring system to predict positive regional nodal status. A ROC curve was then constructed and Youden's index calculated to evaluate the predictive abilities of the various factors. The predictive validity of the scoring system was assessed on the testing sample. DFS estimates were established using the Kaplan-Meier method, and differences in survival between patient subgroups within the training group were evaluated using log-rank tests. Multivariate analyses of DFS were performed using Cox proportional regression models. The significance level was set at 5% in each analysis.

## References

[1] Monson JR, Weiser MR, Buie WD, Chang GJ, Rafferty JF, Buie WD, Rafferty J, Standards Practice Task Force of the American Society of C, Rectal S (2013). Practice parameters for the management of rectal cancer (revised). Diseases of the colon and rectum.

[2] Bujko K, Sopylo RL (2007). Local Excision after Radio(chemo)therapy for Rectal Cancer: is it Safe?. Clinical Oncology.

[3] Garcia-Aguilar J, Pollack J, Lee SH, Anda EHD, Mellgren A, Wong WD, Finne CO, Rothenberger DA, Madoff RD (2002). Accuracy of endorectal ultrasonography in preoperative staging of rectal tumors. Diseases of the Colon & Rectum.

[4] In Ja P, Y Nancy Y, Skibber JM, Rodriguez-Bigas MA, Barry F, Sa N, Chung-Yuan H, Chang GJ (2013). Comparative analysis of lymph node metastases in patients with ypT0–2 rectal cancers after neoadjuvant chemoradiotherapy. Diseases of the Colon & Rectum.

[5] Bedrosian I, Rodriguez-Bigas MA, Feig B, Hunt KK, Ellis L, Curley SA, Vauthey JN, Delclos M, Crane C, Janjan N (2004). Predicting the node-negative mesorectum after preoperative chemoradiation for locally advanced rectal carcinoma. Journal of Gastrointestinal Surgery.

[6] Lezoche E, Baldarelli M, Lezoche G, Paganini AM, Gesuita R, Guerrieri M (2012). Randomized clinical trial of endoluminal locoregional resection versus laparoscopic total mesorectal excision for T2 rectal cancer after neoadjuvant therapy. British Journal of Surgery.

[7] Lezoche G, Baldarelli M, Mario G, Paganini AM, Sanctis A, De Bartolacci S, Lezoche E (2008). A prospective randomized study with a 5-year minimum follow-up evaluation of transanal endoscopic microsurgery versus laparoscopic total mesorectal excision after neoadjuvant therapy. Surgical Endoscopy.

[8] Nair R, Siegel EM, Yeatman TJ, Malafa MP, Marcet J, Shibata D (2008). 216 Long-Term Results of Transanal Excision After Neoadjuvant Chemoradiation for t2 and T3 Adenocarcinomas of the Rectum. Journal of Gastrointestinal Surgery.

[9] Chan AKP, Alfred W, Daryl J, John H, Donald B, Douglas J (2005). Posttreatment TNM staging is a prognostic indicator of survival and recurrence in tethered or fixed rectal carcinoma after preoperative chemotherapy and radiotherapy. International Journal of Radiation Oncology Biology Physics.

[10] Perez RO, Habr-Gama A, Proscurshim I, Campos FG, Kiss D, Gama-Rodrigues J, Cecconello I (2007). Local excision for ypT2 rectal cancer--much ado about something. Journal of Gastrointestinal Surgery.

[11] Ri-Sheng Z, Hui W, Zhi-Yang Z, Qian Z, Mulholland MW (2014). Restaging of locally advanced rectal cancer with magnetic resonance imaging and endoluminal ultrasound after preoperative chemoradiotherapy: a systemic review and meta-analysis. Diseases of the Colon & Rectum.

[12] Juefeng W, Kaitai L, Ji Z, Guichao L, Zhen Z (2015). Implications for selecting local excision in locally advanced rectal cancer after preoperative chemoradiation. Oncotarget.

[13] Callender GG, Das P, Rodriguez-Bigas MA, Skibber JM, Crane CH, Krishnan S, Delclos ME, Feig BW (2010). Local excision after preoperative chemoradiation results in an equivalent outcome to total mesorectal excision in selected patients with T3 rectal cancer. Annals of Surgical Oncology.

[14] De Felice F, Izzo L, Musio D, Magnante AL, Bulzonetti N, Pugliese F, Izzo P, Di Cello P, Lucchetti P, Izzo S, Tombolini V (2016). Clinical predictive factors of pathologic complete response in locally advanced rectal cancer. Oncotarget.

[15] Smith FM, Chang KH, Sheahan K, Hyland J, O'Connell PR, Winter DC (2012). The surgical significance of residual mucosal abnormalities in rectal cancer following neoadjuvant chemoradiotherapy. The British journal of surgery.

[16] Chang HC, Huang SC, Chen JS, Tang R, Changchien CR, Chiang JM, Yeh CY, Hsieh PS, Tsai WS, Hung HY (2012). Risk factors for lymph node metastasis in pT1 and pT2 rectal cancer: a single-institute experience in 943 patients and literature review. Annals of Surgical Oncology.

[17] Deborah S, Ulf G, Martin J (2013). Predicting lymph node metastases in early rectal cancer. European Journal of Cancer.

[18] Simha V, Kapoor R, Gupta R, Bahl A, Nada R (2014). Mucinous adenocarcinoma of the rectum: a poor candidate for neo-adjuvant chemoradiation?. Journal of Gastrointestinal Oncology.

[19] Qiu HZ, Wu B, Xiao Y, Lin GL (2011). Combination of differentiation and T stage can predict unresponsiveness to neoadjuvant therapy for rectal cancer. Colorectal Disease.

[20] Bratland A, Vetrhus T, Grøholt KK, Ree AH (2010). Preoperative radiotherapy in rectal signet-ring cell carcinoma - magnetic resonance imaging and treatment outcome: Report of six cases. Acta Oncologica.

[21] Kim NK, Kim YW, Min BS, Lee KY, Sohn SK, Cho CH (2009). Factors associated with local recurrence after neoadjuvant chemoradiation with total mesorectal excision for rectal cancer. World Journal of Surgery.

[22] Tong T, Yiqun S, Sanjun C, Zhen Z, Yajia G (2015). Extramural depth of rectal cancer tumor invasion at thin-section MRI: predicting treatment response to neoadjuvant chemoradiation. Oncotarget.

[23] An C, Huh H, Han KH, Kim MJ, Kim NK, Kim H, Lim JS (2015). Use of Preoperative MRI to Select Candidates for Local Excision of MRI-Staged T1 and T2 Rectal Cancer: Can MRI Select Patients With N0 Tumors?. Diseases of the Colon & Rectum.

[24] Sobin LH, Gospodarowicz MK (2009). C. W. UICC TNM Classification of Malignant Tumors.

